# Experimental demonstration of a non-resonant hyperlens in the visible spectral range

**DOI:** 10.1038/ncomms8201

**Published:** 2015-05-22

**Authors:** Jingbo Sun, Mikhail I. Shalaev, Natalia M. Litchinitser

**Affiliations:** 1 Department of Electrical Engineering, University at Buffalo, The State University of New York, Buffalo, New York 14260, USA

## Abstract

A metamaterial hyperlens offers a solution to overcome the diffraction limit by transforming evanescent waves responsible for imaging subwavelength features of an object into propagating waves. However, the first realizations of optical hyperlenses were limited by significant resonance-induced losses. Here we report the experimental demonstration of a non-resonant waveguide-coupled hyperlens operating in the visible wavelength range. A detailed investigation of various materials systems proves that a radial fan-shaped configuration is superior to the concentric layer-based configuration in that it relies on non-resonant negative dielectric response, and, as a result, enables low-loss performance in the visible range.

## Abstract (web summary)

Metamaterial hyperlenses convert evanescent waves into propagating ones, thereby beating the diffraction limit. Sun *et al*. present a non-resonant waveguide-integrated hyperlens designed around a radial fan structure that provides imaging at visible wavelengths with low losses.

## Graphical abstract

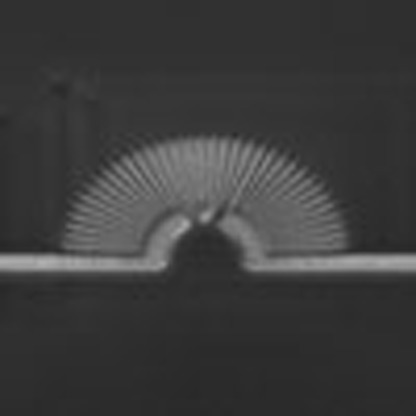

As the resolution of conventional optical systems is limited by diffraction, the visualization of features smaller than the wavelength of the illuminating light requires the development of new imaging techniques. Such techniques would revolutionize many fields ranging from clinical diagnostics and single molecule spectroscopy to nanoscale lithography. For instance, it was shown that an ability to visualize nanoscale structures might be critical for early detection of various cancers, such as ovarian cancer, which is the fifth leading cause of death due to cancer in women^1^, or adenocarcinoma in patients with chronic gastroesophageal reflux disease^2^.

Metamaterial-based optics was predicted to solve the problem of diffraction-limited resolution of conventional optical components^3^,^4^,^5^,^6^,^7^,^8^,^9^,^10^. One of the most promising approaches to high-resolution applications is the so-called hyperlens^11^,^12^,^13^,^14^. Hyperlenses overcome the diffraction limit by transforming evanescent waves, responsible for imaging subwavelength features of an object, into propagating waves. Once converted, those formerly decaying (evanescent) components commonly lost in conventional optical imaging can now be collected and transmitted using standard optical components^11^,^12^. A hyperlens is a curved (cylindrically or spherically shaped) hyperbolic metamaterial with negative dielectric permittivity along the radial direction and a positive permittivity along the tangential direction. To date, the most commonly used design to realize such a hyperbolic metamaterial is the concentric-ring multilayered structure^13^,^14^,^15^,^16^,^17^,^18^. Drude dispersive dielectric permittivity of metals is always negative below the plasma frequency, which is usually in the deep UV range for metals such as silver or gold. Therefore, the only way to achieve the right indefinite property is to obtain negative permittivity by using resonant negative dielectric permittivity in the direction perpendicular to the metal layer and positive dielectric permittivity along the metal layers by choosing the working frequency close to the plasma frequency^19^,^20^,^21^,^22^. However, the use of resonant structures results in a limited bandwidth and high losses near the resonance. An alternative approach would rely on having negative dielectric permittivity along metal layers (or metal wires) and positive dielectric permittivity in the orthogonal direction^23^,^24^,^25^,^26^,^27^,^28^. As discussed in detail below, this alternative approach has strong potential to enable a low-loss hyperlenses. However, until now, it had only been designed and demonstrated in the microwave frequency range and in acoustics^29^,^30^,^31^, while the challenge of fabricating such structures in the optical frequency range has still not been overcome.

Here, we propose and experimentally demonstrate a non-resonant waveguide-integrated hyperlens with a radially oriented layered structure in the visible frequency range. We perform a detailed investigation of various materials systems and prove that a radial (fan-shaped) configuration is superior to the concentric-layers-based configuration. Indeed, the radial configuration with a negative component of dielectric permittivity orientated along the layers and positive permittivity being normal to the layers relies on non-resonant negative dielectric response and results in a low-loss performance in the visible range. In such a device, the evanescent-wave components of a wave vector from the subwavelength slits are converted into the propagating waves and then out-coupled to the free space in the far field.

## Results

### Optimization of the structure and materials

The dielectric permittivity of a hyperlens is described by an indefinite tensor:


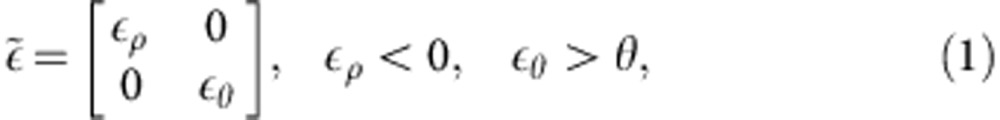


where *ɛ*_*ρ*_ is the permittivity component in the radial direction, which in our case corresponds to the direction along the layers, and *ɛ*_*θ*_ is the azimuthal component of the dielectric permittivity. Therefore, the equi-frequency contour of such a structure in the *ρ*-*θ* plane can be described by refs 18, 30, 31


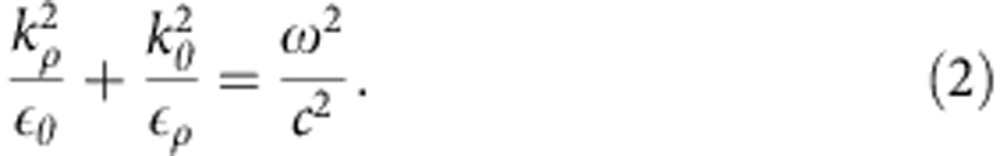


In equation 2, *k*_*ρ*_ and *k*_*θ*_ are the wavevector components along the radial and azimuthal directions, respectively; *c* is the speed of light, and *ω* is the frequency of the incident wave.

**Figure 1:**
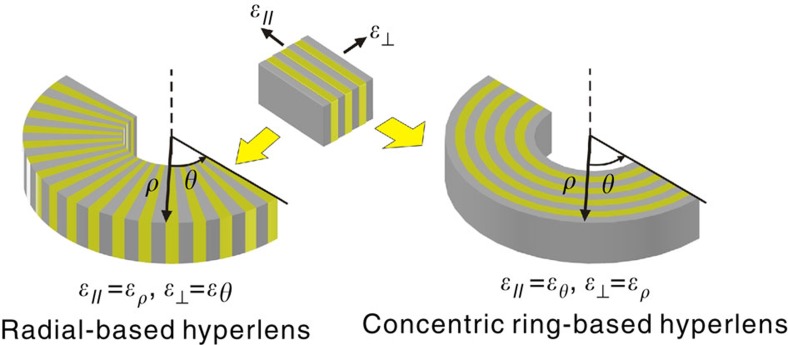
Radial- and concentric ring-based hyperlens realized using multilayered structures of different orientations.

As shown in Fig. 1, the indefinite tensor in equation 1 can be realized in different frequency ranges using metal/dielectric multilayered metamaterial in two orientations: (I) *ɛ*_⊥_=*ɛ*_*θ*_>0, *ɛ*_*//*_=*ɛ*_*ρ*_<0 and (II) *ɛ*_*//*_=*ɛ*_*θ*_>0, *ɛ*_⊥_=*ɛ*_*ρ*_<0, where *ɛ*_*//*_ is the complex dielectric permittivity along the layers and *ɛ*_⊥_ is the complex dielectric permittivity perpendicular to the layers.

Using the Maxwell-Garnett theory, dielectric permittivity tensor components of the multilayered structure can be determined by


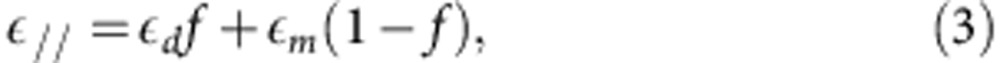



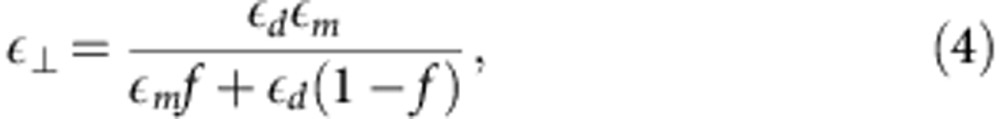


where *f* is the filling ratio of the dielectric material (for example, poly(methyl methacrylate), or PMMA). The frequency dependence of the permittivity of metal can be described by the Drude model as 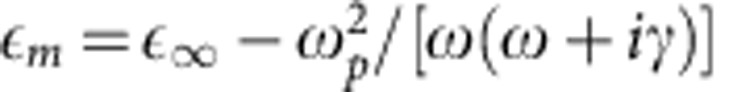, while the permittivity of the PMMA, *ɛ*_*d*_, is nearly frequency independent. According to equation 3, frequency dependence of dielectric permittivity along the layers, *ɛ*_*//*_, is dominated by the properties of metal and is described by the Drude-like model with an effective plasma frequency *ω*_pe_ (corresponding to the wavelength *λ*_pe_) for the given filling ratio *f*. This effective Drude model shows that for *λ*<*λ*_pe_, *ɛ*_r,//_>0 and for *λ*>*λ*_pe_, *ɛ*_r,//_<0. Here, the subscript ‘*r*' denotes the real part of dielectric permittivity. Moreover, the value of *λ*_pe_ increases with the increasing *f* of dielectric material. According to equation 4, frequency dependence of dielectric permittivity perpendicular to the layers is described by the Lorentz-like model, and the real part of dielectric permittivity becomes negative (*ɛ*_r,⊥_<0) at wavelengths below the resonance.

**Figure 2:**
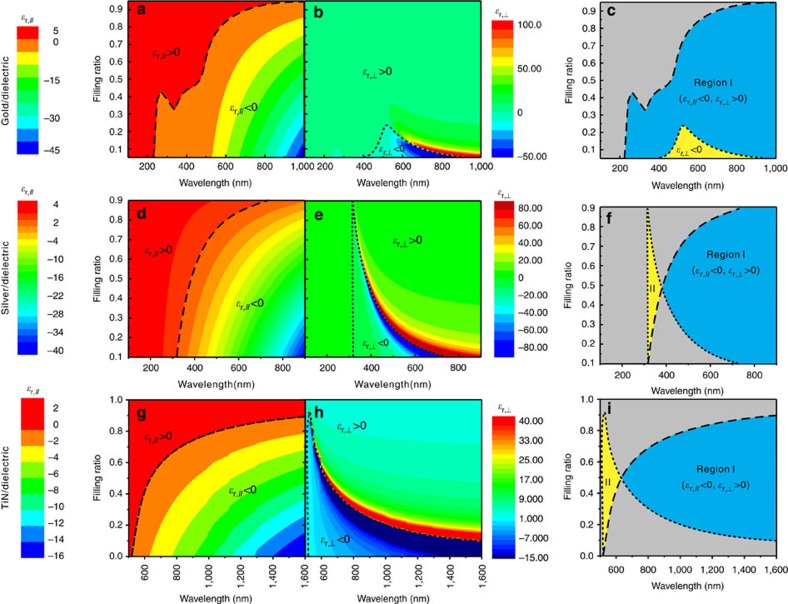
Theoretical analysis of the real part of dielectric permittivity components as functions of filling ratio and wavelength for various materials systems. Real part of parallel (*ɛ*_*//*_) and perpendicular (*ɛ*_⊥_) components of effective dielectric permittivity as a function of filling ratio (*f*) and wavelength (*λ*) for gold/dielectric (**a**–**c**), silver/dielectric (**d**–**f**), and TiN/dielectric (**g**–**i**). The first two columns (figs. **a**, **b**, **d**, **e**, **g**, **h**) show the calculated effective permittivity. The dashed black curves indicate the *λ*_pe_(*f*) for *ɛ*_r,*//*_ in these three cases. Negative *ɛ*_r,⊥_ regions resulting from the Lorentz resonance are enclosed by the dotted black curves. The third column (**c**, **f**, **i**) shows the wavelength regions corresponding to the indefinite permittivity: (I) *ɛ*_⊥_=*ɛ*_*θ*_>0, *ɛ*_*//*_=*ɛ*_*ρ*_<0 shown by the blue colour and (II) *ɛ*_*//*_=*ɛ*_*θ*_>0, *ɛ*_⊥_=*ɛ*_*ρ*_<0 shown by the yellow colour in (**f**, **i**).

Here, we investigate indefinite dielectric properties of three multilayered structures consisting of one of three metallic materials (gold^32^, silver^32^ and TiN^33^), and a dielectric material (*ɛ*_d_=2.1, for example, PMMA or MgF_2_) in different wavelength ranges and with different layer orientations. The real parts of the dielectric permittivity components of such multilayered structures for different filling ratios can be calculated using equations 3 and 4 and are shown in Fig. 2. Figure 2a,d and g, and Fig. 2b,e and h, shows *ɛ*_r,//_ and *ɛ*_r,⊥_ as functions of wavelength and filling ratio for the three combinations, respectively. The dashed black curves indicate the *λ*_pe_(*f*) for *ɛ*_*//*_ in these three cases. Negative *ɛ*_r,⊥_ regions resulting from the Lorentz resonance are enclosed by the dotted black curves. Figure 2c,f and i shows the wavelength regions corresponding to the indefinite dielectric properties.

According to Fig. 2a,b, the Au/dielectric multilayer possesses indefinite properties only in one orientation: *ɛ*_r,//_<0, *ɛ*_r,⊥_>0, which is in the Region ‘I' between the dashed and dotted black curves, and it covers the entire visible range provided that a proper filling ratio is chosen, as shown by the blue colour in Fig. 2c. In contrast, the indefinite property of the Ag/Dielectric and TiN/Dielectric can be obtained in two orientations.

According to the dispersions in Fig. 2d,e,g,h, there are two regions (‘I' and ‘II') located in between the dashed and dotted black curves. In the case of Ag/dielectric multilayer, Region I, corresponding to *ɛ*_r,//_<0, *ɛ*_r,⊥_>0 (blue colour), completely covers the visible range, while Region II, corresponding to *ɛ*_r,//_>0, *ɛ*_r,⊥_<0 (yellow colour), only appears in the ultra-violet (UV) frequency range, as shown in Fig. 2f. Similar to the Ag/dielectric case, in the case of TiN/dielectric, both orientations are possible, but *λ*_pe_ is significantly larger. As shown in Fig. 2i, in particular, Region I, corresponding to *ɛ*_r,//_<0, *ɛ*_r,⊥_>0 for the TiN/dielectric multilayer, covers the range of *λ*>640 nm, shown by the blue colour. Region II, corresponding to *ɛ*_r,//_>0, *ɛ*_r,⊥_<0, is rather narrow and only extends from 510 to 640 nm, shown by the yellow colour. These results are also summarized in Table 1.

From the fabrication view, a multilayered structure with *ɛ*_r,//_>0, *ɛ*_r,⊥_<0 (Region II) is easier to realize. However, as it is shown in Table 1, this orientation corresponds to a limited bandwidth and high losses owing to the resonant nature of negative permittivity response (the imaginary part of the dielectric permittivity components is shown in the Supplementary Fig. 5). For the Ag/dielectric case, Region II only covers the UV frequency range. For the TiN/dielectric multilayer, Region II is broader; however, the material losses are significantly larger than those of Ag (in addition to the losses associated with the resonance). In contrast, based on the data in Fig. 2 and Table 1, a multilayer with *ɛ*_r,//_<0, *ɛ*_r,⊥_>0 (Region I) possesses significantly wider bandwidth (extending over the entire visible range in the cases of Ag or Au) and significantly lower losses owing to the non-resonant origin of the negative dielectric permittivity component *ɛ*_r,//_. These conclusions motivated us to take the challenge of designing and realizing a hyperlens based on *ɛ*_r,//_<0, *ɛ*_r,⊥_>0 orientation. Here, we demonstrate a non-resonant hyperlens with a radially oriented layered structure in the visible frequency range using a combined top-down and bottom-up fabrication approach.

### Design of the waveguide-coupled radial hyperlens

**Figure 3:**
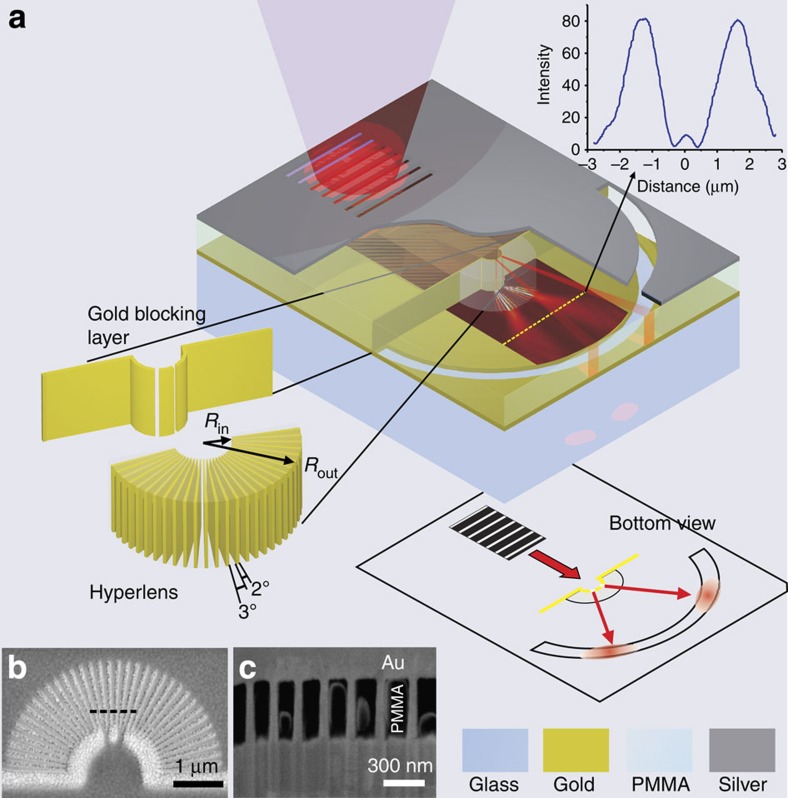
Optical fan-shaped hyperlens with radial layered structure. (**a**) Schematic of the hyperlens integrated with a metal-insulator-metal waveguide. A grating is made on top of the waveguide as an input coupler and a curved slit on the bottom metal layer of the waveguide is used to couple the output beam from the hyperlens into the free space. Inside the waveguide, two nano-slits made in a gold blocking layer are used as the subwavelength objects. The bottom view inset shows the whole integrated optical device from the output side. The inset shows numerical results of imaging of two sub-diffraction-limited slits. (**b**) PMMA pattern including the hyperlens and the blocking layer after the EBL. (**c**) Cross-section (54°) view of the hyperlens structure after the electroplating of gold. This image is taken at the position indicated by the dashed line in **b**. The black part is the PMMA, and the grey part is gold, which is filled into the slots in **b**.

From the above analysis, we chose Au/PMMA materials combination with a PMMA filling ratio of 60%. In this case, the non-resonant indefinite properties (*ɛ*_r,ρ_=*ɛ*_r,//_<0, *ɛ*_r,θ_=*ɛ*_r,⊥_>0) are expected to be observed in the range from 500 to 1,000 nm. The schematic and actual images of the hyperlens that we designed and fabricated in this work are shown in Fig. 3. The hyperlens was composed of 35 pairs of Au/PMMA layers arranged in a fan-like shape and integrated with a metal-insulator-metal (MIM) waveguide on a chip, as shown in Fig. 3a. The entire structure, including the hyperlens itself and a blocking layer with two nano-slits, was first made in a 300-nm-thick PMMA layer placed on top of a gold film using the standard electron-beam lithography technique (including e-beam exposure, development and lift-off steps). As a result, fan-shaped PMMA walls with air slots between them were made as shown in Fig. 3b. Next, gold was filled into these air slots using an electroplating method. Figure 3c shows the cross-section of the hyperlens structure after electroplating. On top of the PMMA, a 300-nm-thick layer of silver was deposited to form the MIM consisting of the top silver layers, the PMMA guiding layer and the bottom gold film. As a result, the hyperlens was directly integrated with the MIM waveguide. Finally, a focused ion beam was used to mill the input and output ports, consisting of a grating coupler and an arc-shaped slot, respectively (fabrication details can be found in the Methods).

To characterize the performance of the waveguide-coupled hyperlens, the transverse-electric mode was coupled into the MIM waveguide using the grating coupler on the top silver layer. This mode impinged on the two nano-slits that served as the subwavelength objects to be imaged and split the original single beam into two beams. Owing to the hyperbolic dispersion property of the hyperlens, evanescent parts responsible for the subwavelength imaging were preserved (along with propagating waves), converted into propagating waves and, finally, magnified by the hyperlens. Consequently, after the transmission through the hyperlens, waves from the two nano-slits were separated enough to be imaged with conventional optics once they were out-coupled through the output arc-shaped port.

Taking into account the validity limits of the effective medium approximation and necessary magnification, the size of the hyperlens (outer/inner radii ratio *R*_out_/*R*_in_) was adjusted to a wavelength range of interest. In particular, the size of each Au/PMMA pair should be much smaller than the wavelength of light on both input and output sides of the hyperlens. In this work, we optimized our samples to be used with a 780-nm source. Here, 780 nm is a free space wavelength that corresponds to an effective wavelength of 860 nm in an MIM waveguide with a 300-nm-thick core (calculation of the effective wavelength can be found in the Supplementary Note 1). The inner and outer radii are 800 and 2,400 nm, respectively, enabling three times magnification. Two nano-slits with widths of 80 nm separated by 250 nm were made in the Au blocking layer and were used as the subwavelengh objects to be imaged. In our design, the width of one Au/PMMA pair at the outer (output) side was about 200 nm, which is still much less than the effective wavelength in the waveguide. At the inner (input) side, each nanoslit size corresponds to almost three pairs of Au/PMMA layers. These considerations ensure that the designed multilayered structure can be approximated as the effective medium. According to equations 3 and 4, the components of the dielectric permittivity for our design are *ɛ*_*ρ*_=−8.93+0.87*i*, *ɛ*_*θ*_=3.65+0.0178*i*, corresponding to very low loss. Numerical simulation results based on the structure with these parameters are supplied in the Supplementary Fig. 4.

### Experimental observation of imaging beyond the diffraction limit

**Figure 4:**
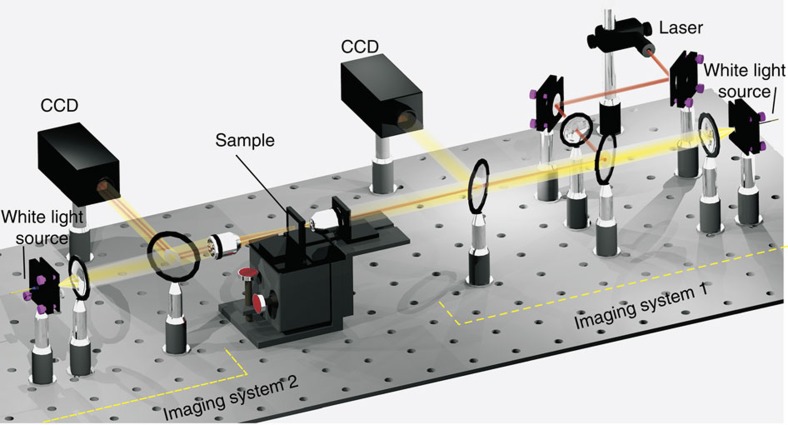
Experimental set-up: twin imaging system. The imaging system 1 consists of a white light source illuminating the sample through an objective and a CCD camera. This system is used to determine the location of the input grating coupler in order to focus the laser beam onto the grating through the same objective. The imaging system 2, consisting of a white light source and a CCD camera is used to locate the output slit and obtain an image of the output beams at the slit.

**Figure 5:**
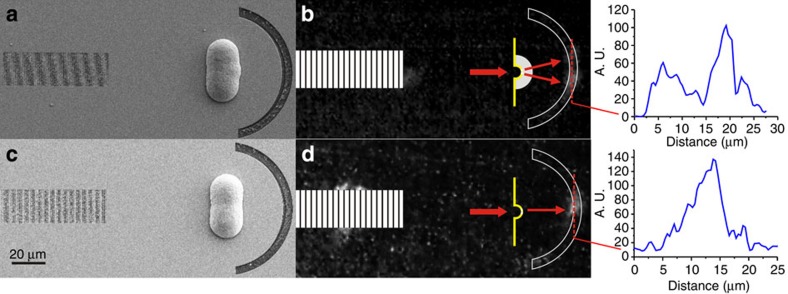
Experimental results. (**a**) SEM image of the entire sample with hyperlens. On the left is the grating coupler used as the input port, and on the right is the output port of the arc-shaped slit. The hyperlens is shown in between the grating coupler and the curved slit. The capsule shaped protuberant ridge is the excess gold, which has expanded out of the PMMA structure during the electroplating. (**b**) Direct visualization at the output port taken using the CCD camera. The panel on the right side shows the profile of the output beams. (**c**) SEM image of the sample in the absence of the hyperlens. (**a**) and (**c**) have the same scale. (**d**) Results without the hyperlens. The panel on the right side shows the profile of the output beams.

To characterize the performance of the waveguide-integrated radial hyperlens, we built a twin imaging system shown in Fig. 4. The location of the input grating coupler (Fig. 5a) was first determined by Imaging System 1 on the right side. Next, a 780-nm laser beam was coupled into the waveguide using the same objective lens of the imaging system to focus it on the grating coupler. The output beams were detected by Imaging System 2 on the opposite side of the sample, ensuring that all incident light coming from the top side was blocked. Using this set-up, we made certain that the power collected by the second imaging system corresponded to light that passed through the waveguide-hyperlens system.

Figure 5b shows the images taken by Imaging System 2. Two output beams are coupled out of the two output slits corresponding to the two beams from the two nano-slits made in the gold blocking layer and then resolved by the hyperlens. The inset of Fig. 5b shows the intensity profile of the two output beams. Finally, a reference sample with two nano-slits on the curved gold blocking layer but without a hyperlens was also fabricated and placed inside the MIM waveguide, as shown in Fig. 5c. Figure 5d shows the image taken by Imaging System 2 without the hyperlens clearly showing only one broad beam coming from the middle parts of the arc-shaped slit, which proves that two beams from the nano-slits cannot be resolved without the hyperlens. The inset of Fig. 5d shows the intensity profile of the output beam.

## Discussion

We proposed a non-resonant magnifying hyperlens operating in the visible frequency range and experimentally demonstrated its sub-diffraction limited imaging at 780 nm. A detailed analysis of various materials systems, including several combinations of metals, such as gold and silver, or alternative plasmonic materials like TiN, and dielectrics, such as PMMA or MgF_2_ indicates that a radial (fan-shaped) configuration is superior to the concentric-layers-based configuration. In particular, experimental characterization of this structure demonstrates a possibility of a low-loss imaging beyond the diffraction limit at visible wavelengths. The proposed radial hyperlens consisting of nanoscale layers of gold and PMMA coupled to a waveguide was fabricated using a combination of electron beam lithography and electro-plating. Finally, theoretical and numerical analysis (that can be found in Supplementary Fig. 7) suggests that optimizing the outer/inner radii ratio and the filling ratio of the dielectric and metal, the hyperlens can be potentially optimized to work in the entire visible range

## Methods

### Sample fabrication

Supplementary Figure 1 shows the schematic of the waveguide-coupled radial hyperlens and the main steps of the sample fabrication process (1)–(5). The insets show the hyperlens and the blocking layer with two nano-slits that serve as the ‘object' to be imaged. In step (1), a PMMA layer was spin-coated on top of the 300-nm-thick gold layer on a glass substrate. Next, in step (2), fan-shaped walls and an inner hyperlens blocking layer were patterned in the PMMA layer using the standard electron beam lithography technique (Vistec EBPG 5,000+). Subsequently, PMMA between the walls was removed during the development and lift-off procedures. The SEM image of the PMMA structure is shown in Supplementary Fig. 2. After the development, the bottom Au layer was exposed in the areas where the PMMA was removed (empty spaces between the fan-shaped PMMA pattern and the blocking layer). These empty parts were then filled with gold using the electroplating method (CHI 660A Electrochemical workstation and Techni-gold 25 es solution, Technic) in step (3). The gold layer under the PMMA was connected to a cathode, while a Pt net was used as the anode. Since the PMMA in the patterned area was removed, the gold layer there was exposed to the solution. After the electroplating, the gaps between the PMMA walls were completely filled with gold, as it is shown in Supplementary Fig. 3. In step (4), a 300-nm-thick silver layer was deposited by E-beam evaporator on the top of the whole structure to create the upper metal layer of the MIM waveguide. Finally, a grating coupler and two half-circular slots were made in step (5) using a focused ion beam system (Zeiss AURIGA CrossBeam Workstation) to create the input and output ports, respectively.

### Experimental set-up

The experimental set-up for characterizing the hyperlens sample is a twin imaging system, as shown in Fig. 4. A White light source (YOKOGAWA AQ4305), a CCD camera (Veho VMS-004 USB microscope without lens) and a × 10 objective lens compose the imaging system 1, which is used to locate the input coupler. A White light source (Thorlab SLS201), a CCD camera (Veho VMS-004 USB microscope without lens) and a 4 × objective lens compose the imaging system 2, which is used to locate the output coupler and catch the image of the output beams. A 780-nm laser is combined into the imaging system 1 by a beam splitter so that it can be coupled into the waveguide through the input coupler. Photos taken by the twin imaging systems on both sides can be found in Supplementary Fig. 6.

## Additional information

**How to cite this article:** Sun, J. *et al*. Experimental demonstration of a non-resonant hyperlens in the visible spectral range. *Nat. Commun.* 6:7201 doi: 10.1038/ncomms8201 (2015).

## Supplementary Material

Supplementary Information Supplementary Figures 1-7, Supplementary Note 1 and Supplementary References.

## Figures and Tables

**Table 1:** Summary of the wavelength ranges corresponding to the indefinite dielectric properties for different materials combinations and hyperlens structures.

**Mechanism**	**Orientation**	**Au/dielectric**	**Ag/dielectric**	**TiN/dielectric**
Non-resonant	*ɛ*_r,//_<0, *ɛ*_r,⊥_>0	Visible	Visible	*λ*>640 nm
Resonant	*ɛ*_r,//_>0, *ɛ*_r,⊥_<0	NA	UV only	510∼640 nm

NA, not applicable; UV, ultra-violet.
