# Supplementary material for: Experimental demonstration of a non-resonant hyperlens in the visible spectral range
Source: Nat Commun. 2015 May 22;6:7201. doi: 10.1038/ncomms8201 (PMC4455119; doi:10.1038/ncomms8201)
Supplement: Supplementary Information — Supplementary Figures 1-7, Supplementary Note 1 and Supplementary References. [file ncomms8201-s1.pdf]

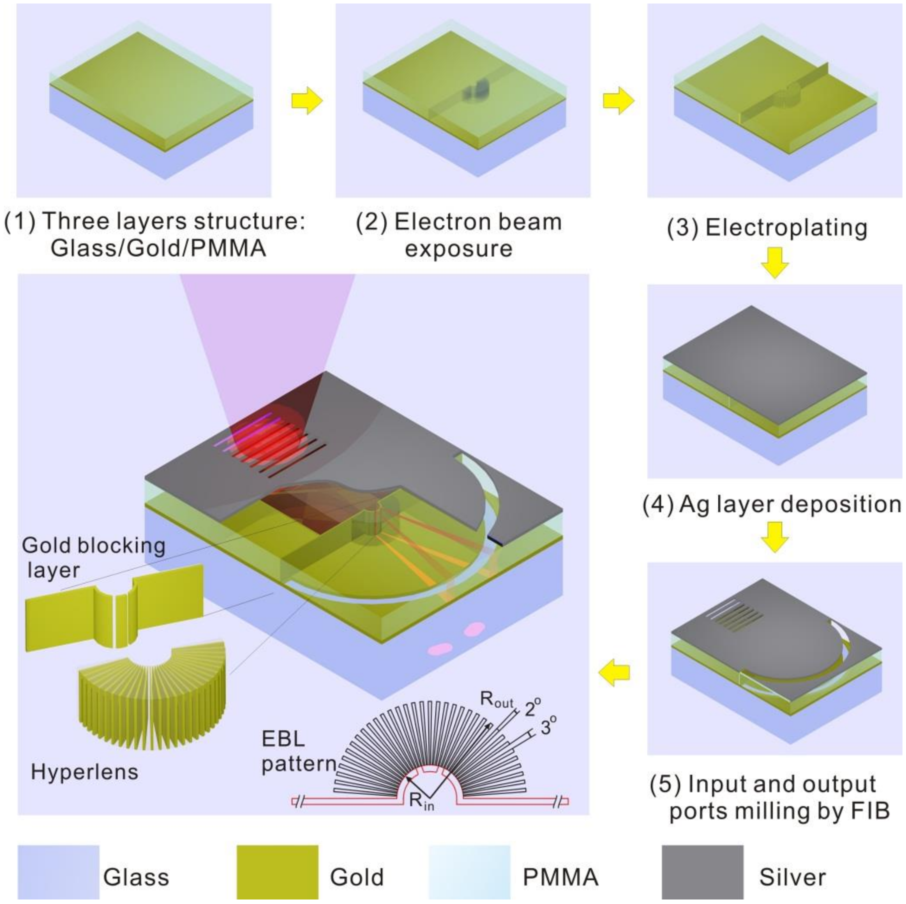

Supplementary Figure 1. The schematic illustration of the proposed device and the fabrication steps. The hyperlens integrated inside a metal-isolator-metal waveguide was made by a combined top-down and bottom-up fabrication approach. Center: schematic of waveguide-integrated radial hyperlens. The insets show the hyperlens and the blocking layer with two nano-slits that serve as the 'object' to be imaged. The fabrication steps are shown in side-figures (1) through (5). To fully demonstrate the resolution property of the hyperlens, we made two kinds of "subwavelength objects" on the surface of the hyperlens: (1) two 80nm wide slits separated by 250nm (center-to center) integrated with an MIM waveguide with 300nm-thick core; (2) two 100nm wide slits separated by 300 nm (center-to-center) integrated with an MIM waveguide with 400nm-thick core.

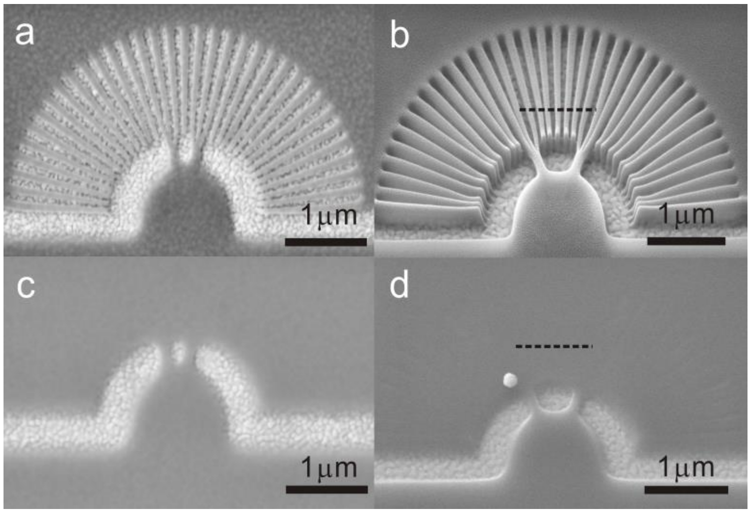

Supplementary Figure 2. Pre-patterned PMMA structures of hyperlens samples and the corresponding reference samples. (a) Hyperlens sample (1) with two 80nm-wide slits separated by 250nm; (b) Hyperlens sample (2) with two 100nm-wide slits separated by 300nm. (c) Reference sample of the hyperlens sample (1); (d) Reference sample of the hyperlens sample (2). The dashed lines show the approximate position of the cross section shown in Supplementary Fig. 3 (b) and (d).

The same steps were repeated for the fabrication of the reference sample that comprised exactly the same structure, waveguide, in- and out-coupling ports, and blocking layer with the slits, but no radial hyperlens. SEM images of the samples are shown in Supplementary Fig. 2. Supplementary Figures 2 a and b show the PMMA structures of the hyperlens samples (1) and (2) patterned using the EBL, respectively. Supplementary Figures 2 (c) and d show the reference samples, which have the same slits sizes as samples (1) and (2), respectively, but no hyperlens structure.

Here we take sample (2) and the corresponding reference sample as an example to show the following fabrication steps. During the electroplating, gold completely filled empty spaces between the PMMA walls and in the space where blocking layers had been pre-patterned; then, excess gold formed a capsule-shaped ridge, as shown in Supplementary Fig. 3 (a) and (c). In order to check the inner structures of the samples, we used the FIB to cut the hyperlens sample and the corresponding reference sample along the dashed lines, as shown in Supplementary Fig. 2 (b) and (d). The cross-sections of the structures are shown in Supplementary Fig. 3 (b) and (d). Supplementary Figure 3 (b) shows vertical layered structure in the hyperlens structure, while no layered structure in the reference sample shown in Supplementary Fig. 3(d).

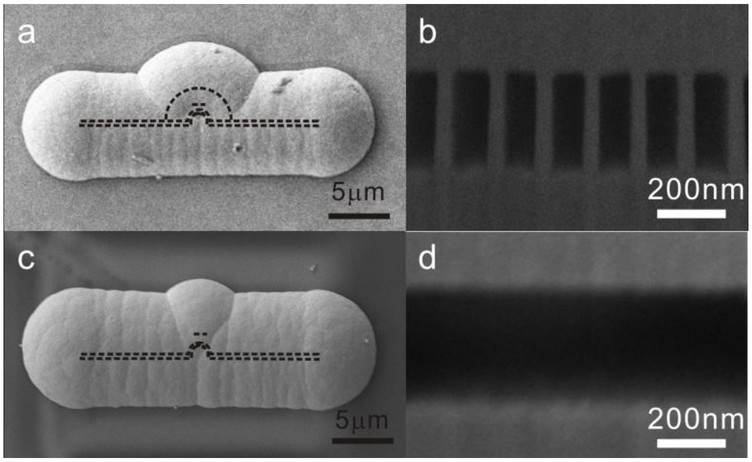

Supplementary Figure 3. Experimental samples after electroplating. Figures 3 (a) and (c) show the structures after the electroplating step, during which, gold completely filled empty spaces between the PMMA walls and in the space where blocking layers had been pre-patterned; then, excess gold formed a capsule-shaped ridge; (b) and (d) show the SEM images of the cross-sections of the hyperlens sample and the reference sample, respectively.

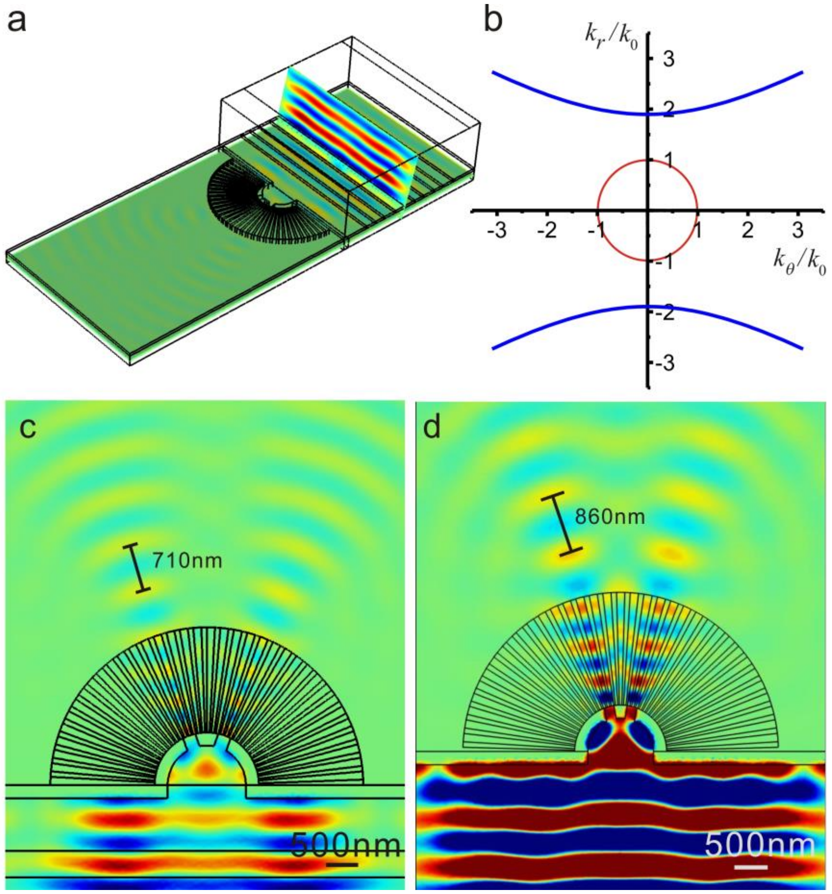

Supplementary Figure 4. Numerical simulations of the proposed device. Simulation setup (a) and the results of light propagation in an integrated system of an MIM waveguide and a hyperlens (b).

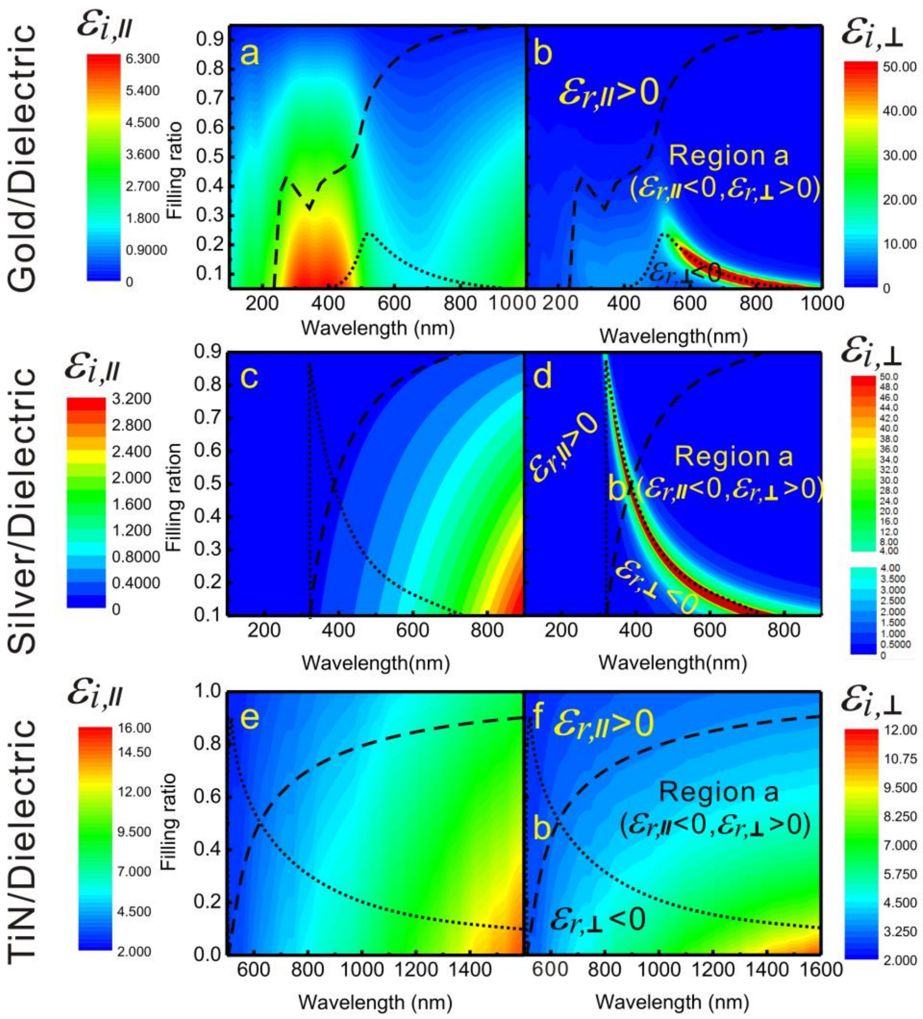

Supplementary Figure 5. Theoretical analysis of losses associated with various materials systems. The figure shows the imaginary part of dielectric permittivity components along the layers ($\varepsilon_{i,//}$) and perpendicular to the layers ($\varepsilon_{i,\perp}$) of the multilayered structure calculated using the Maxwell-Garnett theory for the three cases: Au/dielectric, Ag/dielectric, and the TiN/dielectric. Permittivity data of gold and silver are taken from Supplementary Ref. (2), and TiN permittivity is from Supplementary Ref. (3). As shown in Supplementary Fig. 5(a), (c) and (d), dashed black curves show $\lambda_{pe}(f)$ for $\varepsilon_{//}$ in these three cases. Regions enclosed by the dotted black curves are the ranges of negative $\varepsilon_{\perp}$ corresponding to the resonances, associated with high

resonant losses, as shown in Supplementary Fig. 5(b), (d), and (f). For TiN, resonant loss is much larger than that for Au and Ag since its materials loss is higher in the visible wavelength range. The imaginary parts of $\varepsilon_{//}$ in these three cases are also dominated by the properties of metallic constituents of the multilayers. Supplementary Figure 5(a) shows that $\varepsilon_{//}$ of Au/dielectric multilayer has a relatively large imaginary part around 400nm, which is due to the interband transition of gold in that wavelength range[4]. In Supplementary Fig. 5e, the imaginary part of $\varepsilon_{//}$ is very large in the whole range, caused by relatively high materials loss of TiN.

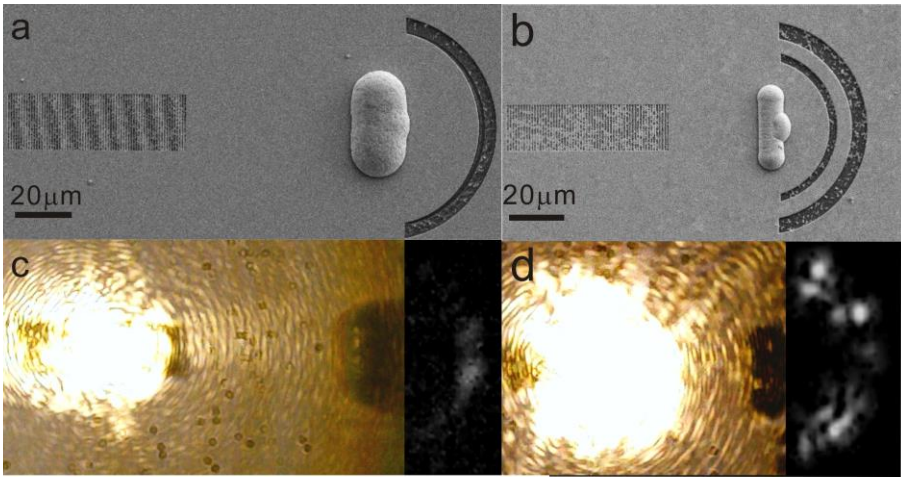

Supplementary Figure 6. Layout of the device. The SEM image of the structure from the top side (a) for sample 1 and (b) for sample (2); (c) and (d) are the measurement results on sample (1) and (2), respectively. Images on the left show the images of the focused beams on the grating from the top side. Images on the right show the images of the output beams resolved by the hyperlens samples. Note that Supplementary Fig. 6 (c) and (d) clearly shows the two output beams resolved by the hyperlens sample (1) and (2), respectively.

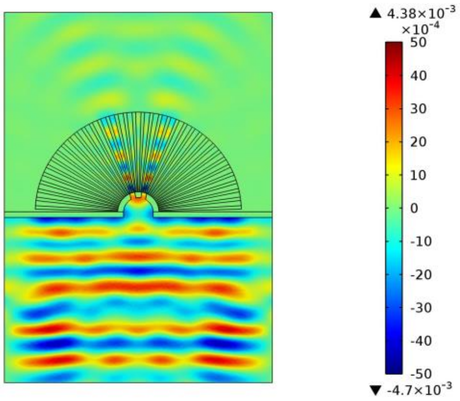

Supplementary Figure 7. Simulation result (H filed) of the hyperlens illuminated with 1064nm light. According to Fig. 2 (a) and (b) in the paper, we can find that the working wavelength can be chosen from the visible to infrared range. Here, we shown a simulation results of the hyperlens with the same filling ratio (0.6) illuminated by 1064nm light. The ratio between the outer radius and the inner radius is 5, which can also give a very good resolution performance. By optimizing the outer/inner radii ratio and the filling ratio of the dielectric and metal, the hyperlens can be optimized to work in the entire visible and near infrared range.

Supplementary Note 1. Numerical simulations:

In the long-wavelength limit, when the feature size of the structure is significantly smaller than the wavelength of incident light, the metamaterial with Au/PMMA alternating layers can be described as an effective uniaxial material. In our design, the radial hyperlens contains 35 pairs of Au and PMMA. Each pair occupies approximately a 5° section. In each pair, Au constitutes about 2°, and the PMMA takes up about 3°. Therefore, the filling fraction of the PMMA is 60%. Using the Maxwell-Garnett theory, we calculated the components of dielectric permittivity along the layers and perpendicular to the layers at 384.6 THz (λ=780nm) to be $\varepsilon_r = -8.93 + 0.87i$ and $\varepsilon_\theta = 3.65 + 0.0178\,i$, respectively. Supplementary Figure 4 (b) shows the EFC of the hyperbolic metamaterial of the hyperlens. A grating coupler was devised on the silver layer[1]. The core thickness *t* of the MIM waveguide was designed to be 300nm and 400nm, which may ensure the fundamental TE mode propagation inside the waveguide. By calculating the propagation constants β of the wave in these two waveguides:

$$\gamma_1 t = m\pi + \arctan\left(\frac{\gamma_2}{\gamma_1}\right) + \arctan\left(\frac{\gamma_3}{\gamma_1}\right),$$

where *m*=1,2,3,… and $\gamma_1^2 = k_0^2\varepsilon_1 - \beta^2$, $\gamma_2^2 = k_0^2\varepsilon_2 - \beta^2$, $\gamma_3^2 = k_0^2\varepsilon_3 - \beta^2$, where ε_1, ε_2 and ε_3 are the permittivity of the PMMA, gold and silver, the effective wavelength in the 300nm core MIM is 860nm and 710nm in the 400nm core MIM, as shown in Supplementary Fig. 4 (c) and (d). Therefore, the slits sizes of both sample (1) and (2) can be treated as subwavelength features. We performed three-dimensional numerical simulations of light propagation in the entire structure consisting of the grating coupler, hyperlens, PMMA layers, and the waveguide using COMSOL Multiphysics™ 4.3b. The results are shown in Supplementary Fig. 4 (a), (c) and (d).

Also, ideally the EFC of the hyperbolic metamaterial should be as flat as possible in imaging applications, our numerical results shown in Supplementary Fig. 4 (c) and (d), confirm an excellent imaging performance of our hyperlens although the EFC shown in Supplementary Fig. 4 (b) is not perfectly flat.

Supplementary References

[1] Born, M., Wolf, E. *Principles of optics* (Cambridge University Press Cambridge UK, 1999)

[2] Palik, E. D. *Handbook of Optical Constants of Solids* (Academic Press CA, 1995).

[3] Naik, G. V., Shalaev,V. M., Boltasseva, A. Alternative plasmonic material: beyond gold and silver. *Adv. Mater.* **25**, 3264–3294 (2013).

[4] Etchegoin, P. G., Le Ru, E. C., Meyer, M. An analytic model for the optical properties of gold. *J. Chem. Phys.* **125** 164705 (2006).
